# Patient Selection for Transcatheter Tricuspid Valve Intervention: Not Too Early, Not Too Late

**DOI:** 10.1016/j.shj.2025.100788

**Published:** 2025-12-19

**Authors:** Jennifer von Stein, Philipp von Stein, Maria C. Alu, Andrea Scotti, Edwin C. Ho, Juan F. Granada, Azeem Latib

**Affiliations:** a Faculty of Medicine and University Hospital Cologne, Department III of Internal Medicine, University of Cologne, Cologne, Germany; b Cardiovascular Research Foundation, New York, New York, USA; c Montefiore-Einstein Center for Heart and Vascular Care, Montefiore Medical Center, Albert Einstein College of Medicine, Bronx, New York, USA

**Keywords:** Transcatheter tricuspid valve intervention, Transcatheter tricuspid valve replacement, Tricuspid regurgitation, T-TEER

## Abstract

Tricuspid regurgitation (TR) is a common but frequently underrecognized condition associated with substantial morbidity and mortality. Long regarded as a mere bystander of left-sided heart disease, TR was often left untreated, contributing to late referrals and poor surgical outcomes. The emergence of transcatheter tricuspid valve interventions has broadened therapeutic options, particularly for high-risk or inoperable patients. However, procedural success and clinical benefit critically depend on appropriate patient selection and timely intervention. This review outlines the evolving landscape of TR management, emphasizing the importance of anatomical and clinical stage-adapted device selection. Key determinants of feasibility and prognosis include right ventricular function and dimensions, TR severity, tricuspid valve leaflet and annular remodeling, and hemodynamic congestion. Advanced imaging modalities and invasive hemodynamics provide incremental value for risk stratification. While tricuspid valve transcatheter edge-to-edge repair (T-TEER) and orthotopic valve replacement are the most widely adopted techniques, direct annuloplasty, heterotopic valve replacement, and coaptation enhancement devices may be more appropriate in anatomically advanced stages. Despite symptomatic improvement and reduced heart failure hospitalizations across different treatment modalities, a survival benefit has yet to be demonstrated. Delayed referral remains a challenge, often precluding repair or even replacement strategies. Dedicated risk models may improve prognostication and guide procedural decision-making. Ultimately, a multidisciplinary approach incorporating multiparametric assessment is essential to identify optimal candidates, guide timing, and personalize therapy. Ongoing trials and long-term outcome data are needed to refine treatment algorithms and clarify the role of early intervention in altering the natural course of severe TR.

## Highlights

• Transcatheter therapies have transformed the management of severe tricuspid regurgitation, yet clinical benefit depends critically on early referral before the onset of advanced right heart failure.
• Integration of multimodality imaging, hemodynamics, and clinical risk scores is essential for optimal patient selection and for tailoring the choice between repair and replacement across the disease spectrum.
• Emerging technologies, including orthotopic and heterotopic valve replacement and coaptation enhancement devices, expand treatment options for anatomically complex patients, but long-term durability, predictors of reverse remodeling, and the role of early intervention remain key areas for future investigation.

## Graphical abstract

Stage-based stratification and device selection in tricuspid regurgitation. Echocardiographic and clinical markers across progressive stages of tricuspid regurgitation guide the choice between transcatheter tricuspid valve repair and replacement. Optimal medical therapy remains essential at all stages. Repair, currently limited to edge-to-edge repair, is often suitable in earlier stages, while orthotopic replacement may also be appropriate depending on anatomical feasibility (left panel). With progressing disease, both repair and orthotopic replacement remain potential options when anatomical requirements are met (middle panel). In very advanced disease stages, neither repair nor orthotopic replacement may be feasible, warranting consideration of heterotopic and coaptation enhancement devices. The illustration is intended as a conceptual framework rather than a definitive treatment algorithm, and device choice should always be individualized. Abbreviations: NYHA, New York Heart Association; RA, right atrium; RV, right ventricle; TR, tricuspid regurgitation.

**Figure:**
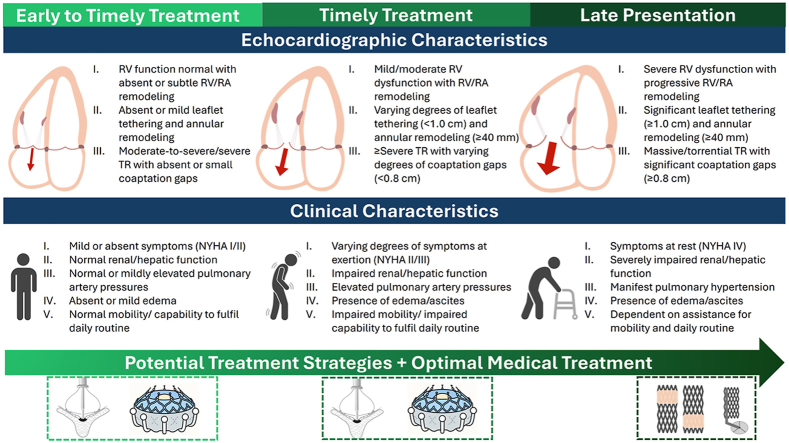

## Introduction

Tricuspid regurgitation (TR) is a common, yet often underrecognized condition with significant impact on both prognosis and quality of life (QOL).^1^^,^^2^ Among individuals over the age of 75 years, severe TR affects approximately 4% of the population.^3^ Despite its growing prevalence, the clinical relevance of TR was long underappreciated, as therapeutic efforts were historically directed toward left-sided heart disease. Medical therapy remains limited to optimal volume management with no proven impact on survival.^4^ Observational evidence further supports the notion that TR confers an independent prognostic burden: increasing TR severity has been independently associated with excess mortality, even after adjustment for pulmonary hypertension (PH), left-sided heart disease, and right ventricular (RV) dysfunction.^5^^,^^6^ In addition to its adverse impact on survival, TR imposes a substantial morbidity burden characterized by recurrent heart failure hospitalizations (HFH), pronounced symptom burden, impaired QOL, and progressive end-organ dysfunction.

Symptoms of TR typically emerge late and lack specificity, often presenting as progressive fatigue, exertional dyspnea, abdominal discomfort, or peripheral edema, and are frequently misattributed to comorbidities. Consequently, diagnosis is often delayed and surgical referral deferred. In-hospital mortality for isolated tricuspid valve (TV) surgery remains high, ranging from 9% to 14%,^7^, ^8^, ^9^ largely reflecting late referral,^9^^,^^10^ and a substantial proportion of patients with severe TR therefore remain untreated.

The magnitude of this unmet clinical need has prompted the development of various transcatheter treatment strategies. Among these, tricuspid valve transcatheter edge-to-edge repair (T-TEER) and orthotopic transcatheter tricuspid valve replacement (TTVR) have become the most widely adopted approaches. In contrast to surgical cohorts, most patients undergoing T-TEER present at an intermediate to late disease stage, with a survival signal observed only in those treated at intermediate stages.^11^ Randomized controlled trials, however, have not shown a mortality benefit, although they consistently demonstrate meaningful improvements in symptoms, QOL, and a reduction in HFH.^12^^,^^13^

Appropriate patient selection and timely treatment remain essential to achieving favorable outcomes. This review summarizes the current evidence on the timing of intervention and outlines transcatheter tricuspid valve intervention (TTVI) treatment strategies tailored to the clinical stage at presentation.

## Etiology and Hemodynamic Impact of Tricuspid Regurgitation

TR was traditionally classified as primary or secondary, reflecting distinct etiologies and anatomical characteristics. Primary TR, accounting for <10% of cases,^14^ results from intrinsic structural valve abnormalities, whereas secondary TR (>90%) arises from RV and/or right atrial (RA) dilation with subsequent tricuspid annular (TA) enlargement and a variable degree of leaflet tethering. The underlying causes include left-sided heart disease (54%), atrial pathologies (24%), pulmonary diseases (17%), and isolated right heart disorders (4%)^15^ (Table 1). Cardiac implantable electronic device (CIED)-induced TR is now recognized as a distinct entity.^16^

**Table 1:** Classification of tricuspid regurgitation etiologies and phenotypes

Causative disease process	Mechanism
Primary TR (∼5-10%)	
Degenerative disease	Prolapse, flail leaflet, myxomatous degeneration
Congenital anomaly	Apical displacement as in Ebstein’s anomaly; leaflet defects (atrioventricular canal, tricuspid hypoplasia)
Acquired (infective endocarditis, trauma, carcinoid, rheumatic, radiation, malignancy)	Leaflet injury, destruction, or infiltration/fibrosis
Secondary TR	
Ventricular secondary (V-STR)	Postcapillary PH due to left-sided ventricular or valvular disease
	Precapillary PH (e.g., chronic lung disease, CTEPH, and PAH)
	Primary RV dysfunction or remodeling (ischemic disease or RV cardiomyopathy)
Atrial secondary (A-STR)	RA/TA dilatation (associated with age, HFpEF, atrial fibrillation)
Lead-related TR (LTR) (∼10%-15%)	
LTR-A (causative)	CIED causative for TR due to impingement, valvular/subvalvular adhesions, perforation; secondary dilatation may be present
LTR-B (incidental)	CIED present without direct interference with the valvular apparatus, not causing TR

Abbreviations: CIED, cardiac implantable electronic device; CTEPH, chronic thromboembolic pulmonary hypertension; HFpEF, heart failure with preserved ejection fraction; PAH, pulmonary arterial hypertension; PH, pulmonary hypertension; RA, right atrial; RV, right ventricular; TA, tricuspid annular; TR, tricuspid regurgitation.

Secondary TR can be further subdivided into atrial secondary TR (A-STR) and ventricular secondary TR (V-STR) phenotypes. A-STR is characterized by predominant RA/TA dilation and is commonly associated with atrial fibrillation (AF), heart failure with preserved ejection fraction, and advanced age,^17^ whereas V-STR typically arises in the context of left-sided heart disease or PH^3^ and is marked by midventricular dilatation and more pronounced leaflet tethering.^18^ The close association between A-STR and AF likely reflects a bidirectional relationship. AF is present in over two-thirds of patients with moderate or severe TR, and its incidence increases with age and the presence of left-sided valve disease.^19^ Interestingly, restoration of sinus rhythm has been associated with a reduction in TR severity in selected cases.^20^ The anatomical and clinical differences between A-STR and V-STR may help explain the more favorable prognosis associated with the atrial phenotype, as supported by previous reports.^21^^,^^22^

Regardless of etiology, TR initiates a uniform hemodynamic cascade driven by chronic volume overload. Early compensatory RV remodeling preserves cardiac output, and patients often remain asymptomatic. With ongoing remodeling, TR worsens, perpetuating a self-reinforcing cycle. As central venous and pulmonary congestion progress, patients develop exertional dyspnea, peripheral edema, abdominal discomfort, and ascites, and require escalating doses of diuretics. In advanced stages, right heart failure (RHF) leads to end-organ dysfunction—most commonly affecting the kidneys^23^ and liver^24^—and presents with fatigue, declining functional capacity, and recurrent HFH, reflecting a transition to low-output syndrome.

The prognosis worsens progressively across increasing stages of RHF,^25^ reflecting the cumulative hemodynamic burden imposed by severe TR. Importantly, TR progression itself carries prognostic significance, with older age, CIED leads, TA dilation, reduced tricuspid annular plane systolic excursion (TAPSE), and prior left-sided valve surgery identified as independent predictors of accelerated progression.^26^ Understanding the hemodynamic trajectory of TR is critical to recognizing when chronic right-sided volume overload begins to manifest as multi-organ dysfunction.

## Current Guideline Recommendations for the Management of Tricuspid Regurgitation vs. Real-World Practice

The new 2025 European Society of Cardiology (ESC)/European Association for Cardio-Thoracic Surgery (EACTS) guidelines on valvular heart disease were only recently released, providing updated recommendations for the management of TR.^27^ Owing to the complexity of TR and its frequent association with comorbidities, both the ESC/EACTS 2025 and American College of Cardiology (ACC)/American Heart Association (AHA) 2020 guidelines emphasize individualized management within a multidisciplinary Heart Team comprising an interventional cardiologist, a cardiac surgeon, a specialist in advanced cardiovascular imaging and periprocedural guiding, as well as additional experts, such as cardiologists with expertise in heart failure, cardiovascular anesthetists, and electrophysiologists.^27^^,^^28^ Key recommendations are summarized in Table 2. A major novelty of the 2025 ESC/EACTS guidelines is the upgrade of TTVI to a Class IIa, Level A recommendation in high-risk patients with symptomatic severe TR despite optimal medical therapy (OMT). Importantly, although new thresholds for defining severe RV dysfunction are suggested, these remain insufficiently validated and primarily serve to guide futility assessment rather than provide strict treatment cutoffs.

**Table 2:** Guideline recommendations for the management of tricuspid regurgitation

Clinical scenario	ESC/EACTS 2025	ACC/AHA 2020
Concomitant left-sided valve surgery	Severe TR: mandatory concomitant repair (Class I). Moderate TR: repair recommended (Class IIa). Mild TR with annular dilatation: may be considered (Class IIb).	Severe TR: mandatory concomitant repair (Class I). Significant annular dilatation (>4.0 cm) or signs of right-sided HF: repair recommended (Class IIa).
Isolated primary TR (symptomatic severe)	Surgery in operable patients without advanced RV dysfunction or pulmonary hypertension (Class I).	Surgery in operable patients (Class IIa).
Isolated primary TR (asymptomatic severe)	Surgery should be considered in patients with RV dilatation/RV function deterioration but without severe LV/RV dysfunction or PH (Class IIa).	Surgery may be considered in patients with progressive RV dilatation/dysfunction (Class IIb).
Isolated secondary TR (severe)	Surgery if symptomatic or in the presence of RV dilatation/dysfunction, provided no severe LV/RV dysfunction or pulmonary hypertension (Class IIa). TTVI if symptomatic despite OMT in high-risk patients without severe RV dysfunction or precapillary PH hypertension (Class IIa).	Surgery only in selected symptomatic patients without PH or left-sided disease (Class IIa).

Abbreviations: ACC, American College of Cardiology; AHA, American Heart Association; EACTS, European Association for Cardio-Thoracic Surgery; ESC, European Society of Cardiology; HF, heart failure; LV, left ventricle/ventricular; OMT, optimal medical therapy; PH, pulmonary hypertension; RV, right ventricle/ventricular; TR, tricuspid regurgitation; TTVI, transcatheter tricuspid valve intervention.

However, despite these clear recommendations, adherence in clinical practice remains limited. In real-world settings, isolated surgical intervention for TR remains rare, with most patients referred at advanced stages. In the United States, isolated TV surgery accounts for less than 5% of all valve procedures.^29^ Among the small proportion of patients who undergo TV surgery, perioperative mortality remains substantial. In a nationwide cohort from Taiwan, in-hospital mortality was 8.7% for isolated TV surgery, with a 5-year all-cause mortality of 42%.^30^ Notably, valve repair was associated with lower risk of long-term mortality, hospital readmissions, and composite adverse outcomes compared with valve replacement—both in isolated and concomitant settings—supporting the general preference for repair over replacement whenever technically feasible.^27^^,^^28^ More recent data from the Society of Thoracic Surgeons (STS) Adult Cardiac Surgery Database reported an operative mortality of 5.6% for isolated TV procedures, with comparable risk between repair and replacement, suggesting improved contemporary outcomes and providing a benchmark for future surgical approaches.^31^

Transcatheter therapies have emerged as promising alternatives, especially in high-risk or inoperable patients. Although registry data and randomized controlled trials suggest favorable safety profiles and outcomes, a survival benefit has yet to be established. While randomized data remain limited, growing observational evidence offers important insights into the real-world performance of surgical and transcatheter approaches. A recent meta-analysis of more than 25,000 patients reported the highest long-term mortality in conservatively treated individuals, whereas surgical and transcatheter approaches yielded similar long-term survival.^32^ Moreover, TTVI was associated with significantly lower short-term mortality and fewer periprocedural complications—including pacemaker implantation, renal dysfunction, and cardiogenic shock—compared with surgery. These findings were further supported by a propensity score-matched cohort of 1143 patients. Between 2016 and 2020, the use of T-TEER increased from 2% to 67%, reflecting its rapid clinical adoption. Despite this shift, 2-year all-cause mortality remained comparable between T-TEER and surgical repair. However, in-hospital mortality (2.5% vs. 12.5%) and permanent pacemaker implantation (0.0% vs. 12.7%) were significantly lower in the transcatheter group.^33^

The steady increase of TTVI reflects the gap between formal guideline recommendations and the elevated procedural risk observed in late-stage referrals. Collectively, these findings highlight the consistently high long-term mortality across all treatment modalities and reinforce the need for earlier referral.

## Echocardiographic and Clinical Risk Stratification of Tricuspid Regurgitation Treatment and Implications for Timing

While transcatheter therapies for TR have rapidly expanded, their clinical success is largely determined by careful patient selection and appropriate timing. Advanced TR is often accompanied by RV dysfunction and systemic congestion—factors that directly impact procedural success and clinical outcomes. Yet, unlike in left-sided valvular disease, objective thresholds for intervention remain poorly defined. A growing body of evidence highlights the prognostic value of echocardiographic markers, invasive hemodynamics, and clinical variables, which together inform a more individualized risk-benefit assessment (Table 3).

**Table 3:** Summary of key risk stratification parameters in severe tricuspid regurgitation

Parameter	Outcome
Echocardiography/CMR	
TAPSE < 17 mm^34^, ^35^, ^36^	Long-term mortality; 1-y mortality
FAC < 35%^34^	Long-term mortality
RVFWS < 23%^37^	Long-term mortality
3D-RVEF < 45%^38^	1-y mortality
TAPSE/PASP < 0.406 mm/mmHg^39^	1-y mortality
RVFWS/PASP ≤ 0.34%/mmHg^40^	2-y mortality
RVEF (CMR) < 45%^41^^,^^42^	1-y mortality and HFH; all-cause-mortality and HFH
Residual TR < 2+^43^	2-y mortality
RA area > 32.5 cm^2^^44^	2-y mortality
LVEF < 60%^45^	In-hospital mortality
Invasive hemodynamics	
PASP > 46 mmHg^46^	2-y mortality and HFH
mPAP > 28 mmHg^44^	2-y mortality
RAP > 17 mmHg^47^	2-y mortality
PCWP > 19 mmHg^47^	2-y mortality
Clinical assessment	
Age ≥ 70 y^45^	In-hospital mortality
NYHA class III-IV^45^	In-hospital mortality
Daily dose of furosemide ≥125 mg^45^	In-hospital mortality
eGFR <30 mL/min/1.73 m^2^^45^	In-hospital mortality
Elevated NT-proBNP (>2728 pg/mL)^44^	2-y mortality
Elevated total bilirubin^45^	In-hospital mortality
Anemia^45^	In-hospital mortality
Signs of right heart failure (e.g., peripheral edema, ascites, jugular venous distension)^45^	In-hospital mortality
Impaired mobility^45^	In-hospital mortality

Abbreviations: 3D, three-dimensional; CMR, cardiac magnetic resonance; eGFR, estimated glomerular filtration rate; FAC, fractional area change; HFH, heart failure hospitalization; LVEF, left ventricular ejection fraction; mPAP, mean pulmonary artery pressure; NT-proBNP, N-terminal pro-B-type natriuretic peptide; NYHA, New York Heart Association; PASP, pulmonary artery systolic pressure; PCWP, pulmonary capillary wedge pressure; RA, right atrial; RAP, right atrial pressure; RVEF, right ventricular ejection fraction; RVFWS, right ventricular free wall longitudinal strain; TAPSE, tricuspid annular plane systolic excursion; TR, tricuspid regurgitation.

## Right Ventricular Function and Dimensions

Echocardiography remains the imaging modality of choice to assess right heart function and dimensions, with advanced modalities such as three-dimensional (3D) and speckle-tracking echocardiography offering incremental value. More recently, cardiac computed tomography (CT) and cardiac magnetic resonance (CMR) have emerged as complementary tools within a multiparametric imaging strategy.^48^ Still, accurate assessment of RV function in the setting of severe TR remains challenging, as conventional echocardiographic parameters are load-dependent and may overestimate RV function in the presence of chronic volume overload and/or PH.^27^^,^^49^ Therefore, a comprehensive evaluation requires integration of multiple measures offered by different imaging modalities.

In conservatively managed patients with clinically significant TR, several studies have consistently shown that conventional RV function parameters hold independent prognostic value. Summarizing this body of evidence, a recent meta-analysis of over 4000 conservatively treated patients with TR ≥ 2+ demonstrated that both TAPSE, reflecting longitudinal RV function, and fractional area change, reflecting both longitudinal and circumferential function, were inversely associated with all-cause mortality.^34^ Notably, impaired RV free wall strain, representing another regional, longitudinal RV function parameter, identified higher rates of RV dysfunction in patients with significant TR and was predictive of outcomes beyond TAPSE and fractional area change using a cutoff of 23%,^37^ underlining the importance of this additive modality in detecting early and subtle RV involvement.

Advanced imaging modalities such as 3D echocardiography and CMR have furthermore highlighted the prognostic relevance of RV ejection fraction (RVEF), with values < 45% consistently linked to worse outcomes.^38^ RVEF provides incremental prognostic value over conventional two-dimensional echocardiographic measures, as it is independent of geometric assumptions, thus better captures the complex RV anatomy and contraction patterns under pathologic loading conditions by incorporating both longitudinal and circumferential function. Importantly, in patients undergoing T-TEER, CMR-derived RVEF, but not TAPSE, was independently associated with all-cause mortality and HFH.^41^

To overcome the limitations imposed by loading conditions on conventional RV parameters, the concept of RV to pulmonary artery (RV-PA) coupling has emerged as a more integrative measure of RV function. With RV-PA uncoupling, RV contractile reserve is exhausted, and systolic output declines in the presence of increased afterload. Among several noninvasive surrogates, the ratio of TAPSE to pulmonary artery systolic pressure (PASP) has emerged as the most widely used echocardiographic marker.^50^ In patients undergoing TTVI, a TAPSE/PASP ratio below 0.406 was independently associated with increased all-cause mortality.^39^ However, noninvasive estimates of PASP are often unreliable in the context of more advanced TR grades, particularly in patients with large coaptation gaps, due to the equalization of RA and ventricular pressures.^51^ Accordingly, invasive RV-PA coupling assessment using right heart catheter-derived PASP demonstrated superior prognostic discrimination, with an optimal cutoff of 0.387.^52^

Importantly, not only functional RV parameters but also RV size itself influences procedural success in patients undergoing TTVI. In the PASTE registry, a large European Real-World registry comprising over 1059 patients undergoing T-TEER exclusively with the PASCAL system, an RV basal diameter ≥42 mm was independently associated with procedural failure (residual TR > 2+).^53^ Similarly, a recent CT-based analysis identified RV end-systolic length as the only significant anatomical predictor of procedural success, with values > 77 mm linked to higher rates of procedural failure and adverse clinical outcomes.^54^

Overall, these data emphasize the importance of a multimodality imaging approach that integrates functional, volumetric, and hemodynamic parameters to refine patient selection and identify those most likely to benefit from TTVI.

## Anatomical Criteria for Procedural Success

### TR Severity and Coaptation Gap

Accurate quantification of TR severity remains a critical yet inherently complex task, owing to the dynamic nature of secondary TR, the noncircular and frequently elliptical regurgitant orifice, and the variability in loading conditions. Current recommendations advocate for a multimodality and multiparametric, integrative approach to TR assessment, balancing qualitative, semiquantitative, and quantitative findings to enable accurate and reproducible TR grading.^55^ Recognizing that many patients present with advanced disease despite being classified as “severe,” an expanded grading scheme, including the categories “massive” and “torrential” TR, was introduced to better capture the full spectrum of TR and to enhance procedural outcome stratification.^56^ Subsequent studies confirmed the incremental prognostic value of this expanded grading scheme.^57^

Among the anatomical parameters most predictive of procedural success, especially in T-TEER, baseline TR severity and coaptation gap dimensions play a central role, as both directly impact device feasibility and effective leaflet approximation. Both massive/torrential TR and a coaptation gap of >8 mm were identified as independent predictors of procedural failure following T-TEER,^53^ with further evidence suggesting that an anteroseptal and central jet is more feasible to address and therefore associated with superior procedural outcomes.^58^

Beyond baseline TR severity, residual TR has emerged as a powerful prognostic marker following TTVI. Data from the Euro-TR registry, the largest multicenter European cohort of patients undergoing T-TEER, demonstrated that residual TR ≤ 2+ was associated with improved 2-year survival and greater clinical benefit compared to patients with higher grades of residual TR.^43^ More recent data, albeit from smaller cohorts, suggest that even more ambitious procedural targets, such as residual TR ≤ 1+, may translate into further survival benefit.^59^ Consistent with these findings, the TRIGISTRY registry, an international multicenter retrospective cohort including both T-TEER and annuloplasty procedures, reported a stepwise improvement in 2-year mortality with lower degrees of residual TR.^60^

As maladaptive RV remodeling remains a key driver of late mortality in severe TR, inducing reverse remodeling through effective TR reduction should be considered a fundamental therapeutic goal. A recent imaging-based study combining 3D echocardiography and CMR in 253 patients treated with either transcatheter repair or replacement showed that those with residual TR ≤ 1+ showed more pronounced reverse remodeling, although this did not translate into a 1-year survival difference.^61^ In a separate 3D echocardiography-based study examining T-TEER patients, the extent of reverse RV remodeling was strongly associated with improved 2-year survival, underscoring its prognostic relevance.^62^ While TR reduction remains the primary procedural target, reverse remodeling may serve as a clinically meaningful surrogate to guide postprocedural assessment and improve long-term risk stratification. Still, larger prospective studies are warranted to validate these findings.

### Valve Morphology and Tricuspid Annular Remodeling

TA remodeling and associated leaflet tethering have emerged as critical determinants of procedural success across the spectrum of TTVI. Early surgical studies demonstrated that prophylactic annuloplasty in patients with TA dilation (≥40 mm) undergoing mitral valve surgery effectively prevented TR progression and was associated with improved long-term outcomes.^63^^,^^64^ Subsequent surgical data linked specific leaflet configurations, particularly a tenting area ≥2.8 cm^2^ and a tenting height ≥8 mm, to residual or recurrent TR following tricuspid annuloplasty.^65^^,^^66^ Building on these findings, TTVI registry data have further confirmed the prognostic relevance of leaflet tethering, with a tenting height ≥10 mm emerging as an independent predictor of procedural failure.^53^^,^^67^

Emerging evidence has highlighted the prognostic relevance of TV leaflet configuration in T-TEER. While a trileaflet TV morphology is typically assumed, up to one-third of patients exhibit a quadricuspid anatomy.^68^ This anatomical variant has been independently associated with procedural failure, irrespective of baseline TR severity, coaptation gap width, or jet location.^69^ Notably, this association appears specific to leaflet-based repair techniques, as procedural outcomes following direct annuloplasty remain unaffected by leaflet configuration,^70^ which can be attributed to the fundamentally different anatomical targets of these approaches.

Careful evaluation is warranted in patients with transtricuspid CIED leads to accurately characterize the underlying mechanism, as a lead may be causative for TR or incidental without direct interaction with the valve leaflets. Lead-related TR is relatively common, most frequently due to leaflet impingement or adhesions. Procedural success rates with T-TEER can nevertheless be high and comparable to those in patients without leads, although this applies primarily to selected cases.^71^ An individualized case-by-case assessment within the Heart Team is therefore recommended to determine the feasibility of TTVI and/or transvenous lead extraction.

Finally, the GLIDE (gap, location, image quality, density, en-face TR morphology) score, a dedicated risk stratification tool for predicting procedural success of T-TEER, identified five anatomical and technical factors as independent predictors: coaptation gap width, jet location, image quality, chordal density, and en-face TV morphology.^72^ Moreover, higher GLIDE scores were strongly associated with a substantially lower likelihood of achieving residual TR ≤ 1+ in a subsequent analysis.^73^

## Clinical Markers of Congestion and Hemodynamic Parameters

In patients with severe TR considered for TTVI, right heart catheter assessment is recommended to exclude severe PH, typically defined as PASP >70 mmHg.^74^ This is particularly important given the frequent underestimation of PASP by echocardiography in severe TR, owing to the equalization of RA and ventricular pressures. Supporting this, prior data have shown that patients with discordant echocardiographic and invasive PASP estimates experience the poorest 1-year survival, comparable to those with confirmed PH.^51^

More recently, a significant association between invasively measured PASP and clinical outcomes was reported, with PASP ≥ 46 mmHg linked to a markedly increased risk of death or HFH. Remarkably, no significant difference in prognosis was observed between patients with precapillary (pulmonary vascular resistance ≥2 wood units) and postcapillary (pulmonary capillary wedge pressure ≥15 mmHg) PH, with comparable 2-year survival free from HFH (56.3% vs. 57.6%), challenging prior assumptions. The authors attribute these findings to improved patient selection and procedural refinement over time, in contrast to earlier reports demonstrating worse outcomes with precapillary PH.^46^

Beyond afterload, volume status and right-sided congestion have emerged as critical prognostic factors in severe TR. A recent observational study reported that elevated RA pressure (RAP) before intervention independently predicted adverse outcomes after TTVI.^47^ When stratified by volume status, patients with isolated right-sided (RAP ≥17 mmHg) or combined bilateral congestion (RAP ≥17 mmHg and pulmonary capillary wedge pressure ≥19 mmHg) experienced the lowest rates of procedural success and the worst survival. The markedly higher baseline filling pressures and mortality rates observed in this cohort, as compared to the TRILUMINATE Pivotal trial (Trial to Evaluate Cardiovascular Outcomes in Patients Treated with the Tricuspid Valve Repair System),^75^ are likely attributable to the real-world nature of this observational population and further underscore the potential value of a more comprehensive preprocedural optimization.

A recent case series demonstrated that volume overload substantially affects echocardiographic eligibility for T-TEER and that targeted volume management—through intensified diuretic therapy or short-term hospitalization—can reduce coaptation gaps and enable successful T-TEER in patients previously deemed ineligible.^76^ Conversely, in patients undergoing TTVR, excessive diuresis may be detrimental, since CT-based valve sizing is usually performed during screening, and subsequent volume reduction can lead to excessive oversizing at the time of the procedure. Therefore, maintaining comparable weight and volume status at the time of CT planning and on the day of the intervention is crucial.

Collectively, these data highlight the importance of invasive preprocedural hemodynamic assessment to better define volume status, guide targeted decongestion, and enhance procedural success. Prospective studies are needed to confirm these observations and inform standardized optimization strategies.

## Clinical Risk Scores and Implications for Timing

While echocardiographic and hemodynamic parameters remain central to outcome prediction, clinical variables have also been integrated into structured risk models to enhance patient stratification and guide decision-making.

Among the earliest tools applied in the context of TV disease was the European System for Cardiac Operative Risk Evaluation II (EuroSCORE II), a surgical risk model originally developed for left-sided heart procedures. Due to the limited representation of TV surgery in its derivation cohort (∼5%), the EuroSCORE II lacks specificity for isolated TR and was primarily used in the absence of more tailored alternatives.^77^

The STS Predicted Risk of Mortality model for isolated TV surgery incorporates ten preoperative variables: age, sex, prior stroke, need for hemodialysis, left ventricular (LV) ejection fraction, chronic lung disease, New York Heart Association (NYHA) functional class, reoperation, and urgent or emergent status. The resulting clinical risk score ranges from 0 to ≥10, with higher scores associated with a stepwise increase in predicted in-hospital mortality (2% to 34%) and major morbidity (13% to 71%).^78^

To address the suboptimal applicability of general cardiac risk models in patients with isolated TR, the more recently proposed TRI-SCORE was specifically designed to improve risk prediction in this challenging population and incorporates eight clinically relevant parameters: age ≥70 years, NYHA class III or IV, anemia, estimated glomerular filtration rate < 30 mL/min/1.73 m^2^, clinical signs of RHF, impaired mobility, urgent or emergent presentation, and elevated liver enzymes. This score has demonstrated strong discriminatory performance for predicting in-hospital mortality following isolated TV surgery and may serve as a practical tool for individualized risk stratification.^45^

While the STS-Predicted Risk of Mortality and TRI-SCORE offer valuable frameworks for perioperative risk stratification in surgical candidates, their applicability to TTVI remains uncertain. In a recent analysis from the TriValve registry, the TRI-SCORE was externally validated in a large, real-world cohort undergoing TTVI. Although a TRI-SCORE ≥8 was independently associated with increased risk of mortality and HFH, the model demonstrated suboptimal discrimination in this population. Moreover, the prognostic benefit of procedural success, defined as TR reduction ≤2+, was confined to patients with a TRI-SCORE <8, whereas those with higher scores derived limited survival benefit despite technically successful intervention.^79^ These findings are consistent with observations from the TRIGISTRY registry, which demonstrated that both surgical and transcatheter therapies were associated with improved survival compared to conservative management, but only among patients with low or intermediate TRI-SCORE (≤5.)^80^ Consequently, the TRI-SCORE has been acknowledged in the ESC/EACTS 2025 guideline as a dedicated tool for risk stratification in patients with TR, highlighting its value for identifying those most likely to benefit from intervention.^27^

Finally, a machine learning–based survival tree model was introduced to stratify patients undergoing TTVI into three distinct risk clusters using four preprocedural variables: mean PA pressure, RA area, N-terminal pro–B-type natriuretic peptide, and estimated glomerular filtration rate.^44^ This intuitive model identified clusters with markedly different 2-year survival rates, ranging from 85.5% in low-risk to 52.6% in high-risk patients. Compared with conventional surgical scores, it performed at least as well as the TRI-SCORE and outperformed the EuroSCORE II, offering a more pragmatic and procedure-specific approach to risk assessment in TTVI. Although the model was developed and tested in an independent multicenter registry and validated in an external cohort, prospective validation in larger cohorts is warranted.

These findings emphasize the relevance of dedicated risk models in patients undergoing TTVI for severe TR, a population characterized by delayed referral, complex hemodynamics, and multi-organ involvement. Further efforts to develop and validate such tools in large, disease-specific cohorts are warranted to refine patient selection and optimize procedural timing, with artificial intelligence likely to play an increasingly important role.

## Timing and Implications for Device Selection

As transcatheter therapies for severe TR continue to evolve, the feasibility of each approach depends critically on the timing of patient referrals that may limit anatomical suitability and significantly narrow the procedural window. Currently available transcatheter approaches largely mirror established surgical concepts and can be broadly categorized into repair and replacement strategies. Repair techniques include leaflet approximation, such as T-TEER, and direct annuloplasty. Replacement therapies encompass both orthotopic and heterotopic valve implantation, as well as the use of dedicated coaptation enhancement devices. All procedures are typically performed under general anesthesia, with combined fluoroscopic and echocardiographic guidance. Table 4 provides key determinants guiding device selection in TTVI.

**Table 4:** Key criteria for device selection in transcatheter tricuspid valve interventions

Strategy	Favorable	Intermediate	Unfavorable
Leaflet approximation	Small septolateral gap (≤7 mm)^58^ Anteroseptal jet location^58^ Confined prolapse or fail Trileaflet morphology	Septolateral gap >7 mm but ≤8 mm Posteroseptal jet location Nontrileaflet morphology Incidental CIED lead (LTR-B, i.e., without leaflet impingement)	Large septolateral gap >8 mm^53^ Leaflet thickening/shortening (rheumatic, carcinoid), perforation, calcification Dense chordae with marked leaflet tethering >10 mm^53^^,^^67^^,^^72^ Poor echocardiographic leaflet visualization^72^ CIED lead leaflet - interaction (LTR-A) Unfavorable device angle of approach
Annuloplasty	Annular dilatation as primary mechanism of TR Mild tethering (tenting height <0.76 cm, tenting area <1.63 cm^2^, tenting volume <2.3 mL)^66^^,^^81^ Central jet location Sufficient landing zone for anchoring	Moderate tethering (tethering height ≥0.76 cm but <1.0 cm, tenting area >1.63 cm^2^ but >2.5 cm^2^, tenting volume ≥2.3 mL but ≤3.5 mL)^66^^,^^81^ Incidental CIED lead (LTR-B, i.e., without leaflet impingement)	Excessive annular dilatation (exceeding device size) Severe tethering (tethering height >1.0 cm, tenting volume >3.5 mL)^66^^,^^81^ Poor echocardiographic annular visualization Annular proximity of RCA CIED lead leaflet - interaction
Orthotopic valve implantation	Previous surgical repair or bioprosthetic valve replacement Leaflet thickening/shortening (rheumatic, carcinoid) Incidental CIED lead (LTR-B, i.e., without leaflet impingement) Any leaflet morphology	Large gap CIED lead leaflet - interaction (LTR-A)^82^	Excessive annular dilatation (exceeding device size) small right heart chambers^82^ Unfavorable device angle of approach and IVC/TA offset Severe RV dysfunction
Heterotopic valve implantation	Appropriate caval diameters Appropriate intercaval distance (relevant mainly for crosscaval devices) Proximity of the RA to the orifice of the liver veins (<5 mm) No option for direct valve treatment Presence of CIED leads (typically no interaction) Poor or limited TEE imaging windows (procedure feasible with fluoroscopy + TTE only)	Short and tapered SVC Angulated and inversely tapered IVC (Y-shape)	Low RA pressures and a v-wave <15 mmHg (valves will not close). IVC diameter >45 mm for TricValve, and >65 mm for crosscaval devices

Key Criteria for Device Selection adapted from Praz et al.^83^

Abbreviations: CIED, cardiac implantable electronic device; IVC, inferior vena cava; RA, right atrial; RCA, right coronary artery; RV, right ventricle; SVC, superior vena cava; TA, tricuspid annular; TEE, transesophageal echocardiography; TTE, transthoracic echocardiography; TR, tricuspid regurgitation.

## Early to Timely Treatment—Favoring Repair Strategies

### Transcatheter Edge-to-Edge Repair

Among all transcatheter approaches, T-TEER has emerged as one of the most widely adopted techniques, with two dedicated systems currently available: the TriClip (Abbott Vascular) and the PASCAL system (Edwards Lifesciences). The main mechanism of T-TEER relies on direct leaflet approximation by grasping two opposing leaflets, followed by mechanical traction during device closure with an additional indirect annuloplasty effect.^84^

The TriClip, now in its fifth generation, was specifically designed for the TV and features a tailored delivery system, longer clip arms, the ability of independent grasping, and four implant sizes. The prospective, single-arm TRILUMINATE study demonstrated procedural safety and efficacy, with a major adverse event (MAE) rate of 7.1%, residual TR ≤ 2+ in 71% of patients at 1 year, NYHA class ≤ II in 83%, and a mean improvement of 20 ± 1 points in the Kansas City Cardiomyopathy Questionnaire (KCCQ) score.^85^ The 2-year TRILUMINATE Pivotal trial, comparing T-TEER with the TriClip in addition to OMT with OMT alone, confirmed sustained TR reduction to ≤2+ in 84% of device-treated patients, maintained symptomatic benefit, and a significant reduction in HFH (0.19 vs. 0.26 events/patient-year), with no difference in all-cause mortality.^13^ Similar findings were observed in the randomized Tri.FR trial, in which 74.1% of patients in the T-TEER arm met the clinical composite primary endpoint at 1 year compared with 40.6% in the OMT group. T-TEER was associated with significant TR reduction to <4+ in 93.2% of patients, marked improvement in patient-reported outcomes— including a 14.5-point higher KCCQ summary score—and favorable performance on a hierarchical composite of death, valve surgery, HF hospitalization, or ≥15-point KCCQ improvement (win ratio 2.06; *p* = 0.0004).^12^ Notably, the magnitude of TR reduction and improvement in QOL in Tri.FR were comparable to TRILUMINATE, further reinforcing the clinical benefit of T-TEER across different trial settings and patient populations.

The PASCAL system, available in two sizes (P10 and PASCAL Ace), differs from the TriClip in key structural and functional aspects. It features a nitinol frame and a central spacer designed to fill the coaptation gap and reduce leaflet stress, as well as the option for complete elongation to minimize entanglement and allow safe repositioning above the valve plane. In the single-arm, prospective CLASP-TR (Edwards PASCAL TrAnScatheter Valve RePair System in Tricuspid Regurgitation) early feasibility study (EFS), the PASCAL system demonstrated favorable outcomes, with TR ≤ 2+ achieved in 86% of patients, an 18-point KCCQ improvement, NYHA class ≤ II in 92%, and a 1-year MAE rate of 7.7%.^86^ The randomized CLASP II TR pivotal trial, comparing T-TEER with the PASCAL system in addition to OMT with OMT, alone completed enrollment with results expected soon (NCT04097145).

### Direct Annuloplasty

In patients suboptimal for T-TEER—ideally, but not exclusively, those with TA dilatation as the primary mechanism of TR—transcatheter tricuspid valve annuloplasty may be considered. In fact, in a single-center retrospective analysis, the A-STR phenotype was independently associated with higher procedural success rates and superior survival compared to patients with nonatrial TR.^87^

The Cardioband system (Edwards Lifesciences) was the first and only CE-marked device for this approach. The CE mark has since been withdrawn to allow for evaluation of the next-generation Cardioband Fit, which has been under investigation in an ongoing EFS since 2018 (NCT03382457). One advantage of this approach is the preservation of native TV anatomy, which maintains the option for future leaflet-based repair and, in selected cases, may serve as a bridging strategy by reducing TA dimensions and coaptation gap width.^88^ Nonetheless, the considerably longer procedure time compared to T-TEER (202 ± 52 minutes reported in TriBAND^89^) remains a limitation, although the 2-year results of the TRI-REPAIR study were encouraging, demonstrating high technical success (100%), sustained TR reduction to ≤2+ in 72% of patients, and meaningful symptomatic and functional improvements (NYHA class ≤ II in 88%, KCCQ improvement of 14 points).^90^ However, the manufacturer has recently paused further development of the second-generation device.

## Timely Treatment—When to Consider Replacement

TTVR is now an integral part of the treatment landscape for severe TR. Because TTVR devices rely on defined annular size ranges for proper deployment and anchoring, anatomical suitability must be assessed alongside clinical timing. Patients may be unsuitable at both ends of the disease spectrum: those with small right heart and TA dimensions may not accommodate available prostheses, whereas advanced remodeling can likewise preclude safe implantation. Contemporary eligibility analyses consistently show that a substantial proportion of patients are excluded due to annular or RV dimensions outside device-specific ranges, underscoring the need to integrate anatomical and clinical considerations when determining procedural timing.^82^

Several orthotopic TTVR systems are currently under development and clinical evaluation. These include, among others, the Intrepid (Medtronic), LuX-Valve Plus (Ningbo Jenscare Biotechnology), Laplace (Laplace Interventional Inc), VDyne (VDyne, Inc), TriSol (TriSol Medical Ltd), Topaz (TRiCares), and Cardiovalve (Cardiovalve Ltd), differing in access, delivery system, anchoring mechanism, and valve sizes.^91^, ^92^, ^93^

The EVOQUE system (Edwards Lifesciences), the first transfemoral TTVR device to receive Conformité Européenne certification and U.S. Food and Drug Administration approval, comprises a trileaflet bovine pericardial valve mounted on a nitinol frame with nine anchors with valve sizes ranging from 44 to 56 mm. One-year results from TRISCEND demonstrated high procedural efficacy, with near-complete TR elimination (residual TR grade ≤1+ in 98%) and marked symptomatic and functional improvement (NYHA class ≤ II in 93%, KCCQ improvement by 26 points).^94^ Safety was considered acceptable, with a relatively high MAE rate of 30%, primarily driven by severe bleeding. In addition, permanent pacemaker implantation (not included in the predefined composite MAE endpoint) was required in 13% of patients. Of note, patients with a TV anatomy precluding proper device deployment on CT or echocardiography, severe RV or LV dysfunction, and a newly implanted transtricuspid CIED lead (<3 months) were excluded from the trial, alongside other clinical criteria.

The TRISCEND II trial further demonstrated that TTVR with the EVOQUE system, in addition to OMT, was superior to OMT alone for the hierarchical composite primary endpoint. This was primarily driven by improvements in symptoms (NYHA ≥ I class) and QOL (KCCQ ≥10 points), while mortality remained unchanged.^95^ Rates of major bleeding (15.4%) and new pacemaker implantation (24.7%) in patients without pre-existing pacemakers remained substantial and were significantly higher than in the control group. Importantly, TTVR led to a marked reduction in right heart chamber dimensions, almost complete elimination of TR (TR ≤ 2+ in 99%), increases in both RV and LV stroke volume, and improved cardiac output, consistent with RV reverse remodeling. In a TRISCEND II subanalysis, 18-month rates of HFH were significantly lower in patients with massive or torrential baseline TR treated with TTVR compared with OMT alone (23.6% vs. 38.8%; absolute reduction −15.2%), whereas no such benefit was observed in patients with severe TR.^96^

The choice between T-TEER and TTVR remains a central clinical question and should be made on an individualized basis, considering clinical factors—such as the ability to tolerate long-term oral anticoagulation, the risk for new-onset conduction disturbances,^97^ sufficient renal function for contrast-enhanced CT planning, and anatomical considerations, where the likelihood of sufficient TR reduction with repair is low (Table 4). The decision requires balancing the finding that T-TEER, although often associated with residual TR > 2+, nonetheless offers an exceptionally favorable safety profile, even in patients with advanced RV dysfunction. By contrast, TTVR achieves a more complete and durable elimination of TR, including in anatomically complex disease, but at the cost of higher periprocedural risk, including higher mortality, conduction disturbances, bleeding, and acute RHF.^95^

### When Orthotopic Replacement Is Not Feasible—Alternative Treatment Strategies

Orthotopic TTVR is generally not recommended in patients with advanced RV dysfunction due to the risk of acute RHF following abrupt elimination of TR and the consequent increase in effective afterload.^98^, ^99^, ^100^ In this context, T-TEER may not be feasible either: large coaptation gaps, anteroposterior jet location, and annular dimensions exceeding available device sizes were the most common anatomical reasons for ineligibility for T-TEER or TTVR, leaving 23.2% of patients unsuitable for either approach.^101^ Another retrospective, single-center study reported a screen failure rate of ∼60%, again predominantly due to anatomical/procedural limitations, with poor clinical outcomes when treated with OMT alone.^102^ In such cases, heterotopic TTVR may serve as a viable alternative, as anatomical exclusion is uncommon. Moreover, it can also be considered as a bailout option after unsuccessful leaflet-based repair.

### Heterotopic Valve Implantation

In heterotopic TTVR, prosthetic valves are implanted in the superior vena cava (SVC) and/or inferior vena cava (IVC) to prevent regurgitant flow from entering the systemic venous circulation, thereby alleviating venous congestion and its associated symptoms. Importantly, the native TV remains untreated. The CE-marked TricValve system (P + F Products + Features) is a transfemoral system composed of two self-expanding bovine pericardial tissue valves, each specifically designed for deployment in the SVC and IVC, respectively.^103^ Importantly, the procedure does not require transesophageal echocardiography and can be performed under fluoroscopy and transthoracic echocardiography guidance with the patient awake, contributing to its procedural simplicity and making it an attractive option in selected patients.

One-year results from the TricBicaval registry demonstrated high intraprocedural success rates and meaningful clinical improvement, with 82% of patients achieving NYHA class I/II after a median follow-up of 8.8 months. Evidence of decongestion was observed, including a reduction in IVC pressures and improvement of overt RHF signs such as edema and ascites—all in a very high-risk population and with a favorable safety profile.^104^

Beyond bicaval prostheses, early experience with cross-caval heterotopic valve implantation has recently been reported, further broadening the spectrum of heterotopic strategies under investigation.^105^^,^^106^ However, long-term outcome data remain limited, particularly regarding the potential impact on RV function due to increased preload in patients with preexisting RV dysfunction.

### Coaptation Enhancement Devices (Spacer)

To address the significant coaptation gaps frequently observed in patients with ≥severe TR who are not candidates for orthotopic TTVR, dedicated coaptation enhancement devices (“spacers”) have been developed and are currently under investigation in EFS (CroíValve, CroíValve Ltd, Versa Vascular, Versa Vascular Inc, and TriPair, Coramaze).

Among the first of these, the CroíValve system consists of a porcine pericardial coaptation element sutured to a nitinol frame, creating a pericardial skirt against which the native leaflets can coapt, and an internal valve to support diastolic flow. The system is secured via a catheter-based delivery mechanism that positions the device without direct anchoring to the annulus; stability is achieved through a self-expanding nitinol stent deployed in the SVC (NCT05913908).

While this novel approach appears inherently atraumatic due to its minimal direct interaction with surrounding tissue, these devices must still demonstrate consistent efficacy in clinical trials. Several challenges remain, particularly related to the complex native TV anatomy: achieving complete sealing of often oval or irregular regurgitant orifices with a circular device, maintaining stability across variable patient positions in the absence of direct annular anchoring, and avoiding flow obstruction due to potential malpositioning.

## Unanswered Questions and Future Research Directions

Despite substantial progress in the transcatheter treatment of severe TR, several key questions remain unresolved. Although TTVI reliably improves symptoms and reduces HFH, a survival benefit has not yet been demonstrated, and robust long-term data on both clinical efficacy and device durability are still lacking. Considering the demographic shift, lifetime management and strategies that preserve future therapeutic options will become increasingly important.

Reverse RV remodeling—observed primarily in patients with effective TR reduction—has emerged as a desirable therapeutic goal, yet reliable predictors of this “responder status” remain undefined. Whether complete TR elimination, particularly with TTVR, confers long-term benefit or increases the risk of RHF in vulnerable patients is uncertain, and risk factors predisposing to RHF in this setting require further investigation. In addition, hypoattenuated leaflet thickening and valve thrombosis remain major concerns that warrant dedicated study.^107^^,^^108^

Future research should move beyond traditional endpoints and incorporate measures that better capture the systemic burden of TR, including renal and hepatic function, diuretic dosages, and markers of venous congestion. Finally, the potential value of treating asymptomatic patients with severe TR deserves critical evaluation, as early intervention may offer the best opportunity to interrupt the trajectory of progressive RHF and prevent irreversible end-organ damage.

## Conclusion

Transcatheter therapies have reshaped the treatment paradigm for symptomatic severe TR, offering effective options for patients previously considered inoperable. To maximize procedural success and clinical benefit, early referral—particularly in early to intermediate disease stages—is essential. Given the anatomical and clinical complexity of TR and the advanced stage at which many patients still present, care should be centralized in high-volume centers with multidisciplinary expertise and access to the full spectrum of transcatheter therapies. As patient selection increasingly integrates imaging, hemodynamic, and clinical assessment, the development and validation of standardized risk stratification tools will be crucial to enable individualized treatment strategies and improve long-term outcomes.

## Review Statement

The review of this manuscript was managed by Guest Editor Omar Abdul-Jawad Altisent, MD.

## Funding

The authors have no funding to report.

## Disclosure Statement

J.v.S. has received speaker honoraria and travel expenses from Edwards Lifesciences. E.C.H. is a consultant or has served on an advisory board for Abbott, Medtronic, Edwards Lifesciences, Philips, GE Healthcare, Johnson & Johnson Med Tech, Shifamed, Neochord, Nyra, Valgen, Meacor, and Trajectory. A.L. is a consultant on the advisory board of Medtronic, Abbott, Boston Scientific, Philips, Edwards Lifesciences, V-dyne, Shifamed, Trilio, Coramaze, Nyra, Tioga, and NeoChord. The other authors had no conflicts to declare.
